# Prevalence and associated factors of locomotive syndrome in young Japanese adults: a cross-sectional study

**DOI:** 10.1186/s12891-024-07493-z

**Published:** 2024-05-10

**Authors:** Yohei Sawaya, Tamaki Hirose, Sora Onuma, Rina Nakajima, Seiya Fujita, Shiori Muroi, Ryo Sato, Lu Yin, Takahiro Shiba, Kaoru Kobayashi, Tomohiko Urano

**Affiliations:** 1 Department of Physical Therapy, School of Health Sciences, International University of Health and Welfare, 2600-1 Kitakanemaru, Otawara, Tochigi 324-8501 Japan; 2 Nishinasuno General Home Care Center, Department of Day Rehabilitation, Care Facility for the Elderly “Maronie-en”, 533-11 Iguchi, Nasushiobara, Tochigi 329-2763 Japan; 3 Kurosu Hospital, 2650 Ujiie, Sakura, Tochigi 329-1395 Japan; 4 Ikeda Memorial Hospital, 129-7 Morijuku Kitsuneishi, Sukagawa, Fukushima 962-0001 Japan; 5 Rehabilitation Center, Shiobara Spa Hospital of Tochigi Medical Association, 1333 Shiobara, Nasushiobara, Tochigi 329-2921 Japan; 6 Department of Rehabilitation, Yokohama Medical and Welfare Centre Konan, 4-6-20 Konandai, Konan-ku, Yokohama, Kanagawa 234-0054 Japan; 7 Integrated Facility for Medical and Long-term care, Care Facility for the Elderly “Maronie-en”, 533-4 Iguchi, Nasushiobara, Tochigi 329-2763 Japan; 8 Department of Geriatric Medicine, School of Medicine, International University of Health and Welfare, 4-3 Kozunomori, Narita, Chiba 286-8686 Japan

**Keywords:** Japan, Locomotion, Locomotive syndrome, Pain, Young adult

## Abstract

**Background:**

The onset of locomotive syndrome (LS) precedes that of frailty. Therefore, the first step in extending healthy life expectancy is to implement measures against LS in young adults. The aim of this study was to investigate the prevalence of LS and its associated factors in young adults for early detection and prevention of LS.

**Methods:**

The participants of this study comprised 413 university students specializing in health sciences (192 males and 221 females) with an average age of 19.1 ± 1.2 years. All participants voluntarily participated in the study and reported no serious health problems. The presence or absence of LS was evaluated using the stand-up test, two-step test, and the 25-question Geriatric Locomotive Function Scale. Additionally, musculoskeletal assessment (one-leg standing, squatting, shoulder elevation, and standing forward bend), body composition analysis (weight, body mass index, body fat mass, body fat percentage, skeletal muscle mass index (SMI), and phase angle), handgrip strength test, physical activity assessment, and nutritional assessment were conducted. Sex-stratified analyses were performed, comparing groups with and without LS. Factors associated with LS were explored using binomial logistic regression.

**Results:**

Of the 413 young adults studied, 86 individuals (20.8%) were found to have LS. When stratified by sex, LS was observed to have a considerably higher prevalence in females (55, 24.9%) than in males (31, 16.1%). In males, the notable differences between the groups with and without LS were observed in one-leg standing and phase angle, whereas in females, differences were identified in body fat mass, body fat percentage, SMI, musculoskeletal pain, and handgrip strength. Two types of binomial logistic regression analysis revealed that the inability to perform one-leg standing was associated with LS in males, while the presence of musculoskeletal pain and a high body fat percentage were identified as factors associated with LS in females.

**Conclusions:**

One in five young adults were found to have LS in this study, underscoring the necessity for early intervention and LS health education. Furthermore, effective management of musculoskeletal pain is also crucial.

**Supplementary Information:**

The online version contains supplementary material available at 10.1186/s12891-024-07493-z.

## Background

Locomotive syndrome (LS), first proposed in 2007, is a condition characterized by a decline in motor skills, such as standing and walking, due to musculoskeletal disorders [1, 2]. As LS progresses, motor skills continue to deteriorate, ultimately resulting in the need for long-term care [2]. In recent years, active research on LS has reported its associations with negative outcomes such as future falls, new cases requiring long-term care, and mortality [3, 4].

In 2022, the Japanese Medical Science Federation published the “Declaration of the Medical Society for Overcoming Frailty and Locomotive Syndrome” [5]. Frailty is defined as “a state of increased vulnerability to various stresses due to a decline in physiological reserve capacity with aging” [6]. The Declaration defines both LS and frailty as (i) conditions characterized by decreased functionality, leading to an increased risk of impaired healthy life expectancy and the need for long-term care, and (ii) conditions that can be prevented or improved through appropriate measures. Consequently, in order to overcome LS and frailty, medical professionals must collaborate to contribute to the health and longevity of the population and strive to maintain individuals’ physical activity levels.

With regard to the relationship between LS and frailty, it is posited that a mild functional decline is initially identified as LS, and that the onset of LS precedes that of frailty [7]. Specifically, in a wide age range including young individuals, a minor functional decline in the musculoskeletal system is initially identified as LS, which gradually becomes more severe and leads to frailty among older adults [5]. Therefore, implementing preventative measures against LS in younger adults could be considered the first step towards extending healthy life expectancy. Existing research has shown that the prevalence of LS is 21.7–25.0% among individuals under 40 years of age [4], and 14.1–21.7% among university students [8, 9], indicating that a certain proportion of young adults also exhibit signs of LS. However, there is a notable lack of research specifically addressing the prevalence and factors of LS among individuals in their teens and twenties. Factors associated with LS in middle-aged to older adults include sex [4, 10, 11], body mass index (BMI) [4, 10, 12], body fat percentage [13], pain [10], muscle strength [4, 10], physical activity [12], breakfast intake [12], nutrition [13] and posture [14]. However, it remains unclear if the same factors are associated with LS in young adults.

Considering the aforementioned information regarding LS in middle-aged to older adults, the objective of this study was to investigate the prevalence and associated factors of LS in young adults, with the aim of facilitating early detection and prevention of LS. In young adults, there are clear sex differences in the skeletal muscle mass and muscle strength [15]. In addition, with regard to body size, both thinness and obesity are problematic, although the rate of emaciation tends to be higher among young females [16]. Considering these sex-specific differences, we hypothesized that the factors associated with LS in young adults are sex-specific. To this end, we collected a sufficient sample size to examine the prevalence of LS and its associated factors and conducted sex-stratified analyses.

## Methods

### Study design and participants

**Fig. 1:**
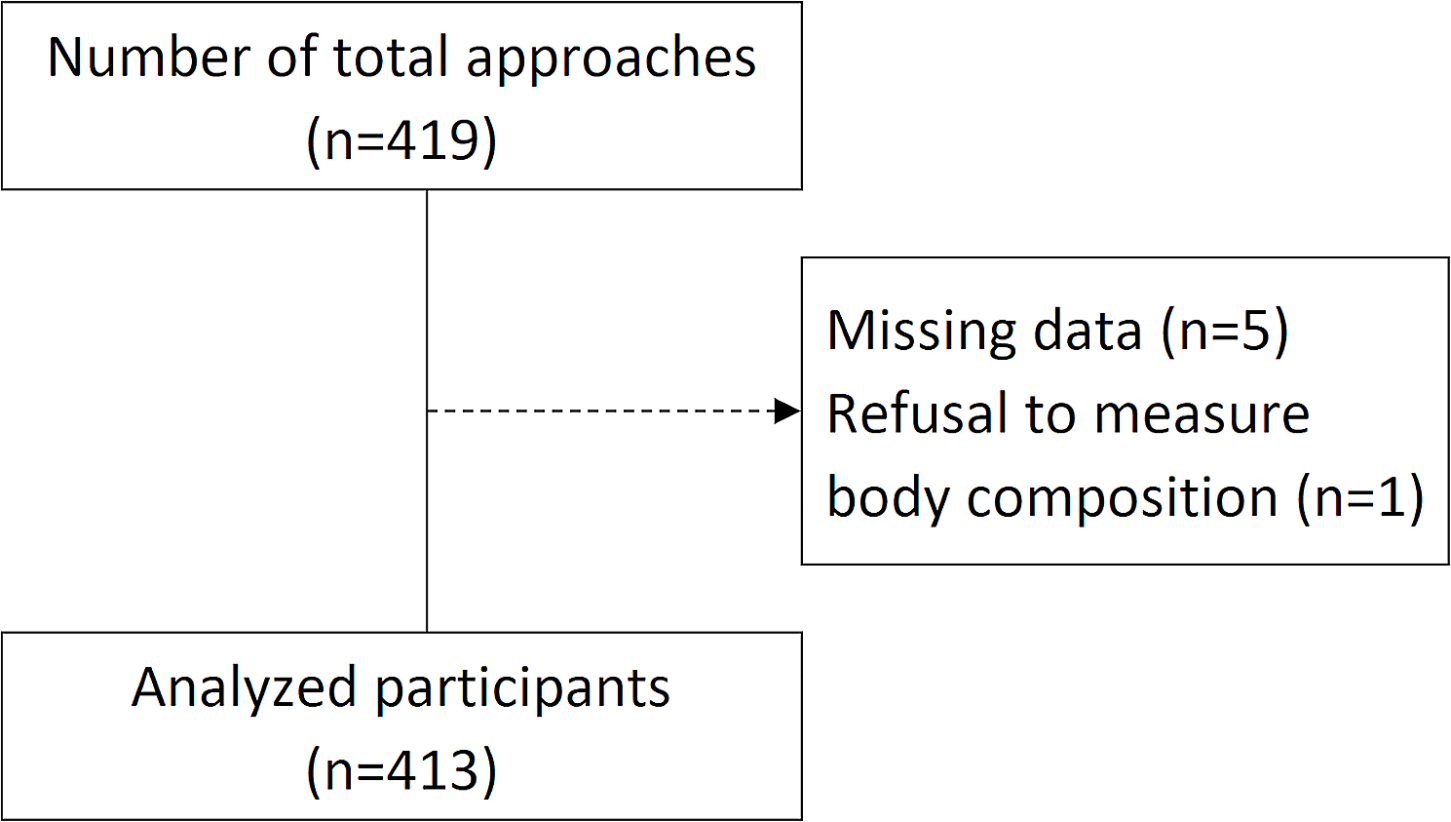
Participant enrollment flowchart

The present study was conducted as a cross-sectional investigation from April to July 2023. Participant recruitment was carried out through a segment of the lecture program, university-wide bulletin postings, and verbal announcements. The study cohort consisted of 419 students enrolled in a health sciences university in Japan. All participants voluntarily participated in the study. Following the exclusion of 5 participants with missing data and 1 participant who declined to participate in the body composition assessment, a total of 413 participants (mean age 19.1 ± 1.2 years, 192 males and 221 females) were included in the analysis (Fig. 1). None of the participants exhibited pain or physical symptoms that could impede the administration of the tests; in other words, none of them exhibited any serious health issues.

### LS

The stand-up test, two-step test, and the GLFS-25 were administered to assess the presence of LS among the participants. The stand-up test assessed the ability to stand up from a 40-cm height using one leg, a 30-cm height using both legs, and a 20-cm height using both legs. Participants were considered successful if they could stand up without using momentum and maintain the standing position for 3 s after standing up. The 40-cm single-leg stand up test was considered successful if the participant could stand up using each leg [2, 17]. In the two-step test, participants took two steps with the longest possible stride from the starting line, and the distance to the position of the toes where they stopped was measured. The maximum value of two measurements was taken as the representative value, and the two-step test score was calculated by dividing the measured length by the height (cm) [2, 17]. The 25-question Geriatric Locomotive Function Scale (GLFS-25) is a self-administered and comprehensive measure consisting of 25 items with a minimum score of 0 and a maximum score of 100, with higher scores indicating severe impairment [2, 17, 18]. Musculoskeletal pain was defined as scoring 1 or more on any of questions 1–4 of the GLFS-25. These three tests are valid and reliable, and their most remarkable feature is the ability to consistently quantify mobility in all age groups, ranging from relatively healthy younger to middle-aged populations to already disabled older adults [18–20]. In this study, the stage of LS according to each test was determined based on the criteria set forth by the Japanese Orthopaedic Association [4, 17] as follows.

Stage 1

Stand-up test: unable to stand up from a 40-cm-high seat using either leg, but able to stand up from a 20-cm-high seat using both legs.

Two-step test: from 1.1 to less than 1.3.

25-question GLFS: from 7 points to less than 16 points.

Stage 2

Stand-up test: unable to stand up from a 20-cm-high seat using both legs, but able to stand up from a 30-cm-high seat using both legs.

Two-step test: from 0.9 to less than 1.1.

25-question GLFS: from 16 points to less than 24 points.

Stage 3

Stand-up test: unable to stand up from a 30-cm-high seat using both legs.

Two-step test: less than 0.9.

25-question GLFS: 24 or more points.

The final stage of LS was determined as the stage with the most severe disability among those identified by the three tests for an individual. For example, if an individual had stage 3 of LS according to the 25-question GLFS, stage 2 of LS according to the stand-up test, and stage 2 of LS according to the two-step test, the final stage would be determined as 3 [17].

### Musculoskeletal check

Four tests were conducted for musculoskeletal assessment: one-leg standing, squatting, shoulder elevation, and standing forward bend [21, 22]. One-leg standing was considered successful if the participant could stand on both legs for at least 5 s without swaying. Squatting was considered successful if the participants could squat with their heels on the ground without stopping or falling. Shoulder elevation was considered successful if the participants could raise their upper limbs vertically. Standing forward bend was considered successful if the participants could touch the floor with their fingers while keeping their knees straight. If at least one of these musculoskeletal tests is applicable, it is considered to be indicative of Child LS [21].

### Body composition

Body composition was measured using the multifrequency bioelectrical impedance analysis method (MC-780 A-N, TANITA, Tokyo, Japan). Participants rested for approximately 10 min or more before measurement of the body composition. The assessed items included weight, body mass index (BMI), body fat mass, body fat percentage, skeletal muscle mass index (SMI), and phase angle. BMI was classified as < 18.5 kg/m^2^ and ≥ 25.0 kg/m^2^ [23]. Body fat percentage is the amount of body fat as a proportion of their body weight. SMI was calculated by dividing skeletal muscle mass of the limbs by the square of height (m). The phase angle, a metric indicative of overall physical condition and nutritional status, was determined using the phase angle value for the left side of the body as the representative value, in line with prior research [24, 25].

### Handgrip strength

Grip strength was measured using a Smedley-type grip dynamometer (TKK 5401 Grip-D, Takei Scientific Instruments, Niigata, Japan). Measurements were taken once on each side while standing, and the highest recorded value was considered representative.

### Physical activity

The Japanese version of the International Physical Activity Questionnaire Short Form was administered [26]. Physical activity was categorized as high, moderate, or low based on the established protocol [27].

### Nutrition

The Dietary Variety Score [28] was administered to assess the frequency of consumption (almost every day, 3 or 4 days per week, 1 or 2 days per week, and seldom) for 10 food types. Consistent with previous studies, meat, fish/shellfish, eggs, milk, and soybean products were classified as high-protein foods, whereas low-protein foods included green and yellow vegetables, potatoes, fruits, seaweeds, and fats/oils. The proportion of participants that reported seldom consuming one or more items was calculated for those two food types [29]. Furthermore, breakfast skipping was defined as not consuming breakfast at least once a week [30].

### Habit of crossing one’s legs on sitting

For the question, “During a 60-min period of sitting on a chair, how long do you usually sit with your legs crossed?“, participants were presented with three response options: (i) I never sit cross-legged, (ii) for 5 min, and (iii) for 20 min or more. The selection of option (iii) was considered indicative of having a habit of crossing one’s legs while sitting.

### Statistical analyses

First, we compared the prevalence of and test results for LS between men and women. Second, we performed sex-stratified comparisons of the assessment results between participants with and without LS. The LS-positive group included participants in stages 1 and 2 of LS. The intergroup comparison was performed using an unpaired t-test, Mann-Whitney U test, chi-square test, and Fisher’s exact test. Third, to examine factors associated with LS, two types of binomial logistic regression analysis were performed for each sex. In pattern I, the presence or absence of LS was used as the dependent variable, and all predictors were entered as independent variables and selected using a stepwise method. In pattern II, the presence or absence of LS was used as the dependent variable, and the items that showed significant intergroup differences were used as independent variables; these were selected using the forced entry method. Multicollinearity was checked before the pattern II analysis, and because Pearson’s coefficient for the correlation between body fat mass and body fat percentage in females was 0.946, only body fat percentage was considered an independent variable. For the factors associated with LS in women in both patterns, specifically musculoskeletal pain and body fat mass, a comparison was performed between pain location and segmental fat mass in relation to the presence or absence of LS.

Furthermore, for the binomial logistic regression analysis, post-hoc power analysis was performed using G*Power version 3.1.9.2. The statistical analyses, excluding the power analysis, were performed using IBM SPSS version 25 (IBM Japan, Tokyo, Japan), with a significance level set at 5%.

## Results

A total of 86 participants (20.8%) were found to have LS. When stratified by sex, there were 31 male participants (16.1%) and 55 female participants (24.9%), with a significantly higher prevalence observed among female participants (*p* = 0.038) (Table 1). Overall, 56 participants (13.6%) were found to have LS based on the stand-up test, 2 (0.5%) based on the two-step test, and 36 (8.7%) based on the GLFS-25 (Table 1). Remarkable differences were observed in sex-stratified comparisons of assessment results between participants with and without LS. Male participants had significant differences in one-leg standing (*p* = 0.003) and phase angle (*p* = 0.045), whereas female participants exhibited significant differences in body fat mass (*p* = 0.044), body fat percentage (*p* = 0.009), SMI (*p* = 0.044), musculoskeletal pain (*p* = 0.001), and handgrip strength (*p* = 0.011) (Table 2).

**Table 1:** Results of locomotive syndrome tests and musculoskeletal assessment

	Total (*n* = 413)	Male (*n* = 192)	Female (*n* = 221)	*P* value
Presence of LS	86 (20.8)	31 (16.1)	55 (24.9)	0.038
LS stage 1	83 (20.1)	31 (16.1)	52 (23.5)	0.035
LS stage 2	3 (0.7)	0 (0)	3 (1.4)	
Stand-up test				
Stage 1	56 (13.6)	20 (10.4)	36 (16.3)	0.086
Two-step score (cm/height)	1.62 ± 0.14	1.65 ± 0.14	1.59 ± 0.13	< 0.001
Stage 1	2 (0.5)	2 (1.0)	0 (0)	0.216
25-question GLFS score	1 [0–3]	1 [0–3]	2 [0–4]	0.001
Stage 1	33 (8.0)	12 (6.3)	21 (9.5)	0.129
Stage 2	3 (0.7)	0 (0)	3 (1.4)	
Stage 1 and 2	36 (8.7)	12 (6.3)	24 (10.9)	0.116
Musculoskeletal check (Failure rate)				
One-leg standing	22 (5.3)	11 (5.7)	11 (5.0)	0.827
Squatting	46 (11.1)	26 (13.5)	20 (9.0)	0.160
Shoulder elevation	12 (2.9)	6 (3.1)	6 (2.7)	1.000
Standing forward bend	140 (33.9)	80 (41.7)	60 (27.1)	0.002
At least one applicable	189 (45.8)	107 (55.7)	82 (37.1)	< 0.001

The numbers in the table are presented as the mean ± standard deviation, *n* (%), and median [25th–75th percentile]

GLFS: Geriatric Locomotive Function Scale; LS: locomotive syndrome

**Table 2:** Results of measurement values with and without locomotive syndrome

	Male (*n* = 192)			Female (*n* = 221)		
	Non-LS (*n* = 161)	LS (*n* = 31)	*P* value	Non-LS (*n* = 166)	LS (*n* = 55)	*P* value
Age (years)	19.4 ± 1.2	19.0 ± 1.1	0.147	19.0 ± 1.2	18.9 ± 1.1	0.477
Height (cm)	171.1 ± 5.6	173.2 ± 5.8	0.063	157.9 ± 4.9	157.8 ± 5.2	0.919
Body composition						
Body weight (kg)	64.8 ± 9.7	66.6 ± 9.7	0.354	52.8 ± 7.4	54.0 ± 9.6	0.333
BMI (kg/m^2^)	22.1 ± 3.1	22.2 ± 2.8	0.934	21.2 ± 2.7	21.6 ± 3.3	0.290
BMI < 18.5	10 (6.2)	0 (0)	0.370	22 (13.3)	4 (7.3)	0.334
BMI ≥ 25.0	30 (18.6)	4 (12.9)	0.609	15 (9.0)	7 (12.7)	0.440
Body fat mass (kg)	11.4 ± 5.2	12.8 ± 5.9	0.186	15.6 ± 4.8	17.3 ± 6.8	0.044
Body fat percentage (%)	17.0 ± 5.6	18.6 ± 5.5	0.142	29.0 ± 5.2	31.2 ± 6.0	0.009
SMI (kg/m^2^)	8.33 ± 0.81	8.16 ± 0.66	0.281	6.57 ± 0.49	6.41 ± 0.52	0.044
Phase angle (degree)	6.3 ± 0.5	6.1 ± 0.6	0.045	5.2 ± 0.5	5.1 ± 0.5	0.146
Musculoskeletal check (Failure rate)						
One-leg standing	5 (3.1)	6 (19.4)	0.003	6 (3.6)	5 (9.1)	0.147
Squatting	21 (13.0)	5 (16.1)	0.579	14 (8.4)	6 (10.9)	0.592
Shoulder elevation	3 (1.9)	3 (9.7)	0.054	4 (2.4)	2 (3.6)	0.640
Standing forward bend	69 (42.9)	11 (35.5)	0.552	45 (27.1)	15 (27.3)	1.000
At least one applicable	86 (53.4)	21 (67.7)	0.169	63 (38.0)	19 (34.5)	0.748
Musculoskeletal pain	47 (29.2)	13 (41.9)	0.204	56 (33.7)	33 (60.0)	0.001
Handgrip strength (kg)	42.9 ± 6.0	42.2 ± 5.4	0.589	27.5 ± 4.6	25.7 ± 4.0	0.011
Physical activity						
Low	45 (28.0)	12 (38.7)	0.510	20 (12.0)	7 (12.7)	0.861
Moderate	46 (28.6)	8 (25.8)		66 (39.8)	24 (43.6)	
High	70 (43.5)	11 (35.5)		80 (48.2)	24 (43.6)	
Breakfast skipping	92 (57.1)	16 (51.6)	0.693	65 (39.2)	20 (36.4)	0.751
Poor high-protein foods	102 (63.4)	19 (61.3)	0.841	117 (70.5)	40 (72.7)	0.864
Poor low-protein foods	104 (64.6)	25 (80.6)	0.096	114 (68.7)	39 (70.9)	0.866
Habit of crossing legs on sitting	57 (35.4)	6 (19.4)	0.096	36 (21.7)	18 (32.7)	0.106

The numbers in the table are presented as the mean ± standard deviation and *n* (%)

BMI: body mass index; LS: locomotive syndrome; SMI: skeletal muscle mass index

The analysis of LS-associated factors based on binomial logistic regression (pattern I) revealed that inability to perform one-leg standing (β = 2.198, odds ratio = 9.007, *p* = 0.001), high body fat percentage (β = 0.076, odds ratio = 1.078, *p* = 0.039), and inability to perform shoulder elevation (β = 1.854, odds ratio = 6.385, *p* = 0.042) were associated with LS in male participants (Table 3). In female participants, high body fat percentage (β = 0.109, odds ratio = 1.115, *p* = 0.001), presence of musculoskeletal pain (β = 1.112, odds ratio = 3.309, *p* = 0.001), and low SMI (β = -1.131, odds ratio = 0.323, *p* = 0.003) were associated with LS (Table 3). The results of Pattern II are shown in Table S1. Post-hoc power analysis revealed powers of 0.948 and 0.998 for male and female participants, respectively, in pattern I and 0.911 and 0.997 for male and female participants, respectively, in pattern II. Furthermore, female participants with LS exhibited a significantly higher prevalence of back pain (*p* = 0.021) and segmental fat mass of the trunk (*p* = 0.022) (Table 4).

**Table 3:** Factors associated with locomotive syndrome according to binomial logistic regression analysis

**Male**	**β**	**Odds ratio**	**95% CI**	***P*** **value**
One-leg standing (success = 0, failure = 1)	2.198	9.007	2.387–33.977	0.001
Body fat percentage	0.076	1.078	1.004–1.158	0.039
Shoulder elevation (success = 0, failure = 1)	1.854	6.385	1.072–38.043	0.042
**Female**	**β**	**Odds ratio**	**95% CI**	***P*** **value**
Body fat percentage	0.109	1.115	1.044–1.190	0.001
Pain (non = 0, presence = 1)	1.112	3.039	1.578–5.854	0.001
SMI	-1.131	0.323	0.153–0.682	0.003

This binomial logistic regression analysis (Pattern I) was selected using a stepwise method with all predictors presented in Table 1 as independent variables

Dependent variable: Non-LS = 0, LS = 1

Male: Nagelkerke R^2^ = 0.141

Female: Nagelkerke R^2^ = 0.175

CI: confidence interval; LS: locomotive syndrome; SMI: skeletal muscle mass index

**Table 4:** Comparison of pain location and body fat mass in females with and without locomotive syndrome

	Non-LS (*n* = 166)	LS (*n* = 55)	*P* value
Pain Location			
Neck or upper limbs	24 (14.5)	9 (16.4)	0.827
Back, lower back or buttocks	27 (16.3)	17 (30.9)	0.021
Lower limbs	13 (7.8)	9 (16.4)	0.074
Body fat mass (kg)	15.6 ± 4.8	17.3 ± 6.8	0.044
Segmental, arms (kg)	1.2 ± 0.4	1.3 ± 0.5	0.185
Segmental, legs (kg)	6.3 ± 1.7	6.8 ± 2.3	0.112
Segmental, trunk (kg)	8.1 ± 2.9	9.3 ± 4.1	0.022

Values are expressed as mean ± standard deviation and *n* (%)

Pain location is defined as whether or not ≥ 1 points of questions 1–3 of the 25-question Geriatric Locomotive Health Scale

LS: locomotive syndrome

## Discussion

The findings of this study revealed that the prevalence of LS among young healthy adults was 20.8%. The factors associated with LS were reduced balance in males and musculoskeletal pain along with a high body fat percentage in females. One of the strengths of this study is the inclusion of an adequate number of participants, enabling sex-stratified factor analysis and exploration of relevant factors from a wide range of potential endpoints. Furthermore, the observed LS prevalence in this study aligns with previous research, demonstrating consistency with both the prevalence reported in studies of the same age group and the reference values of LS tests [19]. Moreover, the higher LS prevalence among women compared to men, as found in previous studies [8], suggests minimal bias in the present study.

According to a review article, the concept of LS encompasses factors that contribute to the progression of reduced mobility, which include (i) pain, (ii) stiffness, (iii) muscle weakness, and (iv) reduced balance [31]. The present study’s identification of reduced balance in males and musculoskeletal pain in females as factors associated with LS is justified because they align with the LS concept. This is particularly relevant for modern Japanese children who are experiencing an increasing incidence of motor skills disorders, such as poor balance and poor body awareness [21]. Furthermore, musculoskeletal problems among elementary school children tend to be more prevalent in boys than in girls [22], and these issues likely persist into adulthood for affected individuals. In the present study, it was observed that a considerably higher proportion of male participants exhibited at least one musculoskeletal problem compared to their female counterparts. The effectiveness of interventions in alleviating musculoskeletal problems [21] suggests that an early approach targeting balance may contribute to the prevention of LS in young adult males. Furthermore, our research aligns with a previous study that identified musculoskeletal pain as the most contributing factor associated with LS among university students of a similar age group [8]. Moreover, the higher prevalence of musculoskeletal pain in women with bone and muscle-related disorders [32] also substantiates our findings.

The two factors associated with LS in women, namely, pain and body fat, were further analyzed with respect to their anatomical site. The findings revealed that both the location of pain and segmental fat mass are associated with the trunk. To elucidate the underlying reasons for this, we draw upon prior research. First, it has been established that intervertebral disc degeneration progresses from an early age, with intervertebral disc degeneration of the lumbar spine observed in 40% of individuals under the age of 30 years, which suggests that low back pain is a common condition among young adults [33]. Second, women, particularly Asian women, tend to have a higher concentration of body fat in the trunk and abdominal regions. When examining body fat distribution in women by anatomical site, an increase in the trunk area is often observed [34, 35]. Consequently, it can be inferred that the trunk region exhibits heightened sensitivity to both pain and body fat. Therefore, for young women, strategies aimed at preventing dysfunction in the trunk region may contribute to the prevention of LS.

Next, we discuss factors related to LS in young adults and middle-aged to older individuals. LS in middle-aged to older individuals is often caused by diseases such as musculoskeletal disease [2, 17, 31]. In addition to these disease factors, lifestyle deterioration is also considered a factor associated with LS in middle-aged to older individuals [11–13]. In this study of young adults, 68.3% showed poor consumption of low-protein foods, 67.3% showed poor consumption of high-protein foods, 46.7% skipped breakfast, 45.8% had musculoskeletal problems, and 20.3% had low PA. Thus, although many young individuals have poor lifestyles, the low quality of lifestyle was not found as a factor contributing to LS. Instead, existing balance impairment and musculoskeletal pain were extracted as associated factors. An integrated interpretation of previous research and our findings indicates that factors associated with LS can differ between young and middle-aged to older adults. The fact that one in five young adults is affected by LS, and even more when including those at risk, underscores the necessity for early screening, lifestyle modification, and LS health education. Furthermore, 36.1% of young adults experience some form of pain, with a higher prevalence among females. Consultation with an orthopedist for pain management is an important measure in preventing LS.

The present study has some limitations. First, it was conducted at a single university, which may limit the generalizability of the findings. In addition, many students underwent data measurements as part of the lecture program, and although a certain amount of rest time was given before the measurements, it was not possible to control how they spent their time before class. For a wider extrapolation, a multicenter study is necessary. Second, because of its cross-sectional design, the study may not provide strong scientific evidence. Going forward, longitudinal research should unravel causal relationships and pinpoint factors that contribute to the transition from a non-LS status to LS in young adults. Third, there may have been unassessed confounding factors, such as specific diseases and psychological factors, that may have influenced the findings. While there are these limitations, the present study, which analyzed sex-specific factors associated with LS in a sample of 413 individuals, holds the potential to contribute to early prevention and intervention strategies for LS in Japan.

## Conclusions

The prevalence of LS in young adults was found to be 20.8%. The factors associated with LS varied between sexes. In male participants, LS was found to be associated with diminished balance, whereas in female participants, the syndrome was found to be associated with musculoskeletal pain and high body fat percentage. As potential interventions for mitigating LS in young adults, enhancing balance in men and strengthening core muscles in women may be effective. Furthermore, given that 36.1% of the participants reported experiencing some form of pain, seeking consultation with an orthopedic specialist for pain management could be a viable course of action.

### Electronic supplementary material

Below is the link to the electronic supplementary material.

Supplementary Material 1

## Data Availability

The datasets used and/or analyzed during the current study are available from the corresponding author on reasonable request.

## Abbreviations

BMI: Body mass index
GLFS-25: Geriatric Locomotive Function Scale
LS: Locomotive syndrome
SMI: Skeletal muscle mass index
