# Supplementary material for: Prevalence and associated factors of locomotive syndrome in young Japanese adults: a cross-sectional study
Source: BMC Musculoskelet Disord. 2024 May 10;25:366. doi: 10.1186/s12891-024-07493-z (PMC11084025; doi:10.1186/s12891-024-07493-z)
Supplement: Supplementary file 1 — Supplementary Material 1 [file 12891_2024_7493_MOESM1_ESM.docx]

**Table S1.** Factors associated with locomotive syndrome according to binomial logistic regression analysis

| Male | β | Odds ratio | 95% CI | P value |
| --- | --- | --- | --- | --- |
| One-leg standing (success=0, failure=1) | 1.991 | 7.326 | 2.035–26.370 | 0.002 |
| Phase angle | -0.735 | 0.408 | 0.224–1.028 | 0.059 |
| Female | β | Odds ratio | 95% CI | P value |
| Pain (non=0, presence=1) | 1.094 | 2.985 | 1.546–5.765 | 0.001 |
| Body fat percentage | 0.105 | 1.111 | 1.040–1.186 | 0.002 |
| SMI | -0.806 | 0.447 | 0.178–1.119 | 0.085 |
| Grip strength | -0.057 | 0.945 | 0.858–1.041 | 0.250 |

In this binomial logistic regression analysis (Pattern II), the items that showed significant intergroup differences were used as independent variables.

Dependent variable: Non-LS=0, LS=1

Male: Nagelkerke R^2^ = 0.111

Female: Nagelkerke R^2^ = 0.183

CI: confidence interval; SMI: skeletal muscle mass index
